# Dermatologic manifestations in patients with the Hermansky–Pudlak syndrome types 1 and 3

**DOI:** 10.1186/s13023-022-02464-w

**Published:** 2022-07-30

**Authors:** Gabriel Santos Malave, Natalio J. Izquierdo, Nestor P. Sanchez

**Affiliations:** 1 Icahn School of Medicine at Mount Sinai, New York City, NY USA; 2 Department of Surgery, School of Medicine, Medical Sciences Campus, University of Puerto Rico, San Juan, PR USA; 3 Department of Dermatology, Ponce Health Sciences University School of Medicine, Ponce, PR USA; 4 Menonita Hospital, Aibonito, PR USA

**Keywords:** Hermansky–Pudlak syndrome, Albinism, Medical dermatology, Rare disease, Genetics

## Abstract

**Background:**

The Hermansky–Pudlak syndrome (HPS) is a genetically heterogeneous group of diseases characterized by oculocutaneous albinism, bleeding diathesis, and systemic complications. It is the most common genetic disorder in Puerto Rico. These patients are at a significant risk of developing a variety of skin complications and little is known about the prevalence of dermatologic diagnoses in this population.

**Objectives:**

To report dermatologic manifestations in patients with Hermansky–Pudlak syndrome (HPS). Secondary aims include skin concerns, sun protection habits, barriers to dermatologic care, and skin cancer knowledge.

**Methods:**

Cross-sectional study with twenty-nine Puerto Rican patients who carried a clinical diagnosis of HPS type 1 or type 3 through a telephonic questionnaire.

**Results:**

Twenty-nine patients participated with a mean (SD) age of 37.3 (16.8) years and the majority were female (69%). The most common diagnoses were skin cancer (34.5%), acne (34.5%), bacterial skin infections (34.5%), warts (24%), urticaria (17.2%), and psoriasis (17.2%). The most common skin concerns were dry skin (62.1%), hair loss (58.9%), redness (34.5%), moles (31%), and rash (31%). The most common sun protection behavior was wearing a shirt that covers the shoulders (93.1%, often or always) and the least common was wearing a hat (24.1%, often or always). Higher income was significantly associated with being more likely to use sunscreen often or always (OR = 3.38, 95% CI 1.02–11.18, p = 0.04). Those in northern urban areas were significantly less likely to report barriers to dermatologic care (OR = 0.13, 95% CI 0.02–0.76, p = 0.02).

**Conclusions:**

This study provides an important overview of the most common self-reported skin manifestations in patients with HPS. Unfortunately, a high prevalence of cutaneous malignancy was reported. The results stress the need for adequate care and potential interventions to promote sun protection behaviors and skin cancer prevention.

## Key message

High prevalence of cutaneous malignancy suggests a need for interventions in patients with Hermansky–Pudlak syndrome.

## Introduction

The Hermansky–Pudlak syndrome (HPS) is an autosomal recessive disorder characterized by oculocutaneous albinism, bleeding diathesis, and systemic complications [1]. There are ten different HPS subtypes with considerable variability in expression and specific comorbidities including pulmonary fibrosis, colitis, immunodeficiency, chronic kidney failure, and a variety of ocular symptoms depending on the subtype [2]. Pulmonary fibrosis is the leading cause of mortality in patients with Hermansky–Pudlak syndrome type 1 (HPS-1) [1].

Although a rare genetic disorder it is the most common genetic disease in Puerto Rico (P.R.) where it has the highest prevalence worldwide. It is particularly prevalent in the northwestern region, where HPS-1 is the most common subtype with a frequency of 1:1,800 and a carrier frequency estimated to be 1:21 due to a founder mutation affecting a 16-base-pair duplication in exon 15 of the HPS1 gene [3]. The Hermansky–Pudlak syndrome type 3 (HPS-3) is most common in the island’s central mountainous region. It has a frequency of 1:4000 and an estimated carrier frequency of 1:32. HPS-3 is also caused by a founder mutation from a deletion in a genetic region of chromosome 3q24 [4]. The high prevalence of patients with HPS makes it a significant public health problem in PR [2, 3].

Symptoms in patients with HPS are caused by alterations in genes disrupting protein trafficking complexes in lysosome related organelles [5–7]. The albinism is due to dysfunctional trafficking of melanin towards melanosomes resulting in pale skin and light hair [8]. Patients with HPS-3 have darker hair and skin pigmentation suggesting melanogenic enzymes can access melanosomes via an HPS3-independent pathway [8, 9]. Due to the dysfunctional trafficking of melanin, patients with the syndrome are at significant risk of developing skin complications from sun exposure.

A previous study on patients with HPS reported a high frequency of solar keratoses, dysplastic nevi, and basal and squamous cell carcinoma [10]. However, no study has characterized the dermatologic findings in Puerto Rican patients with the syndrome in over 20 years [10]. Furthermore, there are no studies available describing the prevalence of dermatologic pathologies in patients with the syndrome.

Studies on patients with various types of albinism report a lack of adherence to sun protection habits due to the uncomfortable sensation and expense involved in purchasing and using sunscreens [11–13]. In addition, not all patients have access to regular dermatologic care. For these reasons, we aim to describe the dermatologic manifestations and sun protection habits in patients with HPS to better understand their challenges and needs. We also attempt to identify potential risk factors for skin cancer and barriers to dermatologic care.

## Methods

We conducted a cross-sectional analysis of patients with a genetic diagnosis of HPS. This was carried out through a survey done by telephone to adult patients selected from medical records of a private ophthalmology clinic in San Juan, P.R. Inclusion criteria included: a diagnosis of HPS based on genetic linkage analysis, age more than 18 years old, and a working telephone number on file. Exclusion criteria were patients unable to give informed consent and patients who did not have a genotypic diagnosis of the syndrome.

Specific components included demographic information, dermatologic features, previous dermatologic diagnoses, skin complaints, sun protection habits, barriers to receiving dermatologic care, and skin cancer awareness. The survey was independently reviewed by two outside parties including a dermatologist and a statistician with expertise in survey development. Items were revised for clarity to ensure responder comprehension. Dermatologic terms and diagnoses were thoroughly explained to participants when collecting data on dermatologic features and history. Participants were given the opportunity to ask questions if they needed clarification on any item of the questionnaire.

Descriptive statistics were done. Bivariate analysis included chi-squared for paired categorical variables. In addition, odds ratios were derived. For continuous variables, paired t test was used. Statistical significance was defined by two-sided p-value < 0.05. Data analyses was conducted using Stata, version 17.

## Results

### Demographics

A total of thirty-one patients were contacted. Twenty-nine agreed to complete the survey for a response rate of 93.5%. Demographic data is displayed in Table 1. Twenty-five patients (86.2%) had a diagnosis of HPS-1 and four (13.8%) had HPS-3. The majority (69%) were female. Their mean (standard deviation) age was 37.3 ± 16.8 years. Approximately half (52%) had an annual income below $10,000 USD.

**Table 1:** Demographic characteristics of HPS patients

Characteristics	Participants (n = 29)
Sex, N%	
Female	20 (69)
Male	9 (31)
Age, mean (SD)	37.3 (16.8)
Geographic area, N%	
North	12 (41.4)
West	7 (24.1)
South	5 (17.2)
East	4 (13.8)
Civil status, N%	
Single	20 (69)
Married/cohabit	8 (27.6)
Divorced	1 (3.4)
Employment status, N%	
Employed/self-employed	15 (51.7)
Student	7 (24.1)
Unemployed	6 (20.7)
Retired	3 (10.3)
Highest education level, N%	
Elementary school	1 (3.4)
High school diploma	2 (6.9)
Some college	5 (17.2)
College/associate degree	15 (51.7)
Post-graduate	6 (20.7)
Annual family income, N%	
< $10,000	15 (51.7)
$10,000–$20,0000	5 (17.2)
> $30,000	7 (24.1)
Not reported	2 (6.9)
HPS type	
HPS-1	25 (86.2)
HPS-3	4 (13.8)

*N* number

### Self-reported dermatologic features of patients

Hair color of patients ranged from white to light brown with most having blonde hair (82.8%). Patients had a variety of eye colors with most (89.7%) having gray, blue, green, or hazel. Two patients with HPS-1 reported having red-colored eyes and one with HPS-3 had brown eyes.

### Self-reported dermatologic diagnoses and dermatologic concerns

Prevalence of skin diseases are listed in Table 2. The most common skin diseases diagnosed by a dermatologist or other physician were skin cancer (34.5%), acne (34.5%), bacterial skin infections (34.5%), warts (24%), urticaria (17.2%), and psoriasis (17.2%).

**Table 2:** Prevalence of skin diseases in HPS patients

Skin disease, N%	Participants (n = 29)
Acne	10 (34.5)
Bacterial skin infection	10 (34.5)
Warts	7 (24.1)
Urticaria	5 (17.2)
Psoriasis	5 (17.2)
Keloid/hypertrophic scar	4 (13.8)
Rosacea	2 (6.9)
Eczema/dermatitis	2 (6.9)
Fungal skin infection	1 (3.4)
Seborrheic dermatitis	1 (3.4)
Herpes labialis	1 (3.4)
Previous history of malignant or premalignant skin lesions	15 (51.7)
Actinic keratosis	13 (44.8)
Dysplastic nevi	5 (17.2)
BCC	4 (13.8)
SCC	2 (6.9)
BCC and SCC	1 (3.4)
Keratoacanthoma	2 (6.9)
Unknown skin cancer	3 (10.3)

*N* number, *BCC* basal cell carcinoma, *SCC* squamous cell carcinoma

Ten patients (34.5%) were previously diagnosed with skin cancer with the majority (80%) having multiple tumors. Age of onset of the first skin cancer diagnosis ranged from 24 to 51 with an average of 40.6 ± 8.7 years. Among those with a history of skin cancer, four (40%) were diagnosed with basal cell carcinomas (BCC), two (20%) with squamous cell carcinomas (SCC), one with both BCC and SCC (10%), and three (30%) did not remember the specific skin cancer. Tumor site was preferentially in the head and neck (80%), limbs (20%), and trunk (10%) Family history of skin cancer (OR = 8.5, 95% CI 1.25–57.9, p = 0.03) and age (OR = 1.11, 95% CI 1.03–1.20, p < 0.01) were significantly associated with a diagnosis of skin cancer. There was no significant association between age of onset of skin cancer and dermatologic diagnosis or photoprotection habits.

Prevalence of skin concerns are listed in Table 3. The most common were dry skin (62.1%), hair loss (58.9%), redness (34.5%), moles (31%), and rash (31%). Those with daily sun exposure reported a significantly higher number of skin concerns than those without sun exposure (p = 0.037).

**Table 3:** Prevalence of skin concerns in HPS patients

Skin concern, N%	Participants (n = 29)
Dry skin	18 (62.1)
Hair loss	17 (58.6)
Redness	10 (34.5)
Moles	9 (31)
Rash	9 (31)
Greasy skin	8 (27.6)
Facial hair	7 (24.1)
Acne	5 (17.2)
Wrinkles	4 (13.8)
Uneven skin tone	3 (10.3)
Itch	2 (6.9)
Scar	1 (3.4)

*N* number

### Sun protection behaviors

History of sun exposure and frequency of sun protection behaviors are shown in Table 4. Twenty-five patients (86.2%) had a history of sunburn. The most common sun protection behavior reported was wearing a shirt that covers the shoulders (93.1%, often or always) and the least common was wearing a hat (24.1%, often or always). Most patients (86.2%) used sunscreen with some degree of frequency but only seventeen (58.6%) reported doing this often or always when out in the sun. Higher annual income was significantly associated with being more likely to use sunscreen often or always (OR = 3.38, 95% CI 1.02–11.18, p = 0.04).

**Table 4:** Sun exposure and sun protection behaviors of HPS patients

	Participants (n = 29)
Current sun exposure, N%	18 (62.1)
History of sunburn, N%	25 (86.2)
Sunscreen use, N%	
Never	4 (13.8)
Rarely	4 (13.8)
Sometimes	4 (13.8)
Often	2 (6.9)
Always	15 (51.7)
Wear shirt that covers shoulders, N%	
Rarely	2 (6.9)
Often	5 (17.2)
Always	22 (75.9)
Wear a hat, N%	
Never	8 (27.6)
Rarely	9 (31)
Sometimes	5 (17.2)
Often	4 (13.8)
Always	3 (10.3)
Wear sunglasses, N%	
Never	4 (13.8)
Rarely	2 (6.9)
Sometimes	1 (3.4)
Often	1 (3.4)
Always	21 (72.4)
Stay in the shade, N%	
Never	2 (6.9)
Rarely	1 (3.4)
Sometimes	3 (10.3)
Often	5 (17.2)
Always	18 (62.1)

*N* number

The primary reasons for not using sunscreen were its sensation being uncomfortable (48.3%) and costs (31%). The most common reasons for not using sun protective clothing were that it was difficult to use (31%) and uncomfortable (31%). The most common reasons for not avoiding the sun were that it was inconvenient (48.3%), difficult (37.9%), and interrupted with daily activities (31%).

### Skin care practices and perceptions of dermatologic care

Twenty-five patients (86.2%) reported having seen a dermatologist in the past with ten (40%) at least once a year. All patients who had never seen a dermatologist reported the desire to do so. Overall, seventeen (58.7%) stated some difficulty in obtaining dermatologic care. Among these, the most common reasons were time (76.5%) and cost (64.7%) followed by lack of adequate transportation (34.5%) and lack of insurance (24.1%). Those in urban areas were significantly less likely to report having difficulty seeing a dermatologist (OR = 0.13, 95% CI 0.02–0.76, p = 0.02).

### Knowledge of skin cancer

Twenty-six patients (89.7%) examined their skin for the development of any changes on a daily, weekly, or monthly basis. All but one patient agreed having their skin regularly examined would make them feel more secure about their health. Twenty-seven patients (93.1%) were worried of someday developing skin cancer. All patients agreed skin cancer can have grave consequences and that they are at a higher risk than others.

## Discussion

This is the first survey on dermatologic diseases and sun protection habits in patients with HPS. The first aim was to characterize the dermatologic profile of these patients. Like Toro et al. we found a large degree in phenotypic variability even within patients with the same type of HPS [10].

The second aim was to determine the most common self-reported skin diseases and concerns in the HPS population. Skin cancer was the second most reported condition demonstrating the high prevalence in this population. Average age of onset of skin cancer in our sample was 30 years lower than the average for all Puerto Ricans regarding BCC (69.8 years) and SCC (73.5 years) [14]. The most common involved site was the head and neck which is consistent with previous studies in the general Puerto Rican population [14]. Consistent with the study by Toro et. al, our sample also reported actinic keratosis, BCC, SCC, and dysplastic nevi [10]. Melanoma is rare, and we found no reported cases of melanoma, however, cases of amelanotic melanoma in HPS have been previously reported in the literature [15]. This is consistent with findings of patients with albinism in Brazil and several countries in Africa [16–21].

Acne was the most common skin condition in our sample which is consistent with its high prevalence in the general United States (U.S.) population. It is the most common skin condition in the U.S. and prior studies estimate a lifetime prevalence of up to 80% [22, 23]. Within Hispanics in the continental U.S., clinical acne has been shown to be among the three most common diagnoses with a prevalence of 20.7% in one study and 32% in another based on a cohort of Hispanic women [24, 25]. These findings are similar to our sample. Warts were also common in our cohort with an increased prevalence than that found globally (7–12%) and in Hispanic patients in the continental U.S (7.1%) [24].

Interestingly, there was also a high prevalence of psoriasis in our sample. A previous study found the prevalence of psoriasis in P.R. to be increased from the global population at 6.1% [26]. However, our sample had an even larger prevalence potentially supporting evidence of a relationship between psoriasis and decreased melanocytes and melanin [27, 28].

It is also of particular importance that several patients reported a diagnosis of keloids. This is contrary to previous studies which indicated that people with albinism do not develop keloids and supports recent studies describing their presence in patients with albinism [29–31]. The study by Kiprono et al. [30], found that patients with albinism are equally affected by keloids as the general population. Our results support their findings that genetic susceptibility could play a more important role in the development of keloids than the amount of skin pigment.

Our third aim was to assess sun protection behaviors. The annual mean ultraviolet index in P.R. is higher than all the continental U.S. [32]. The incidence of skin cancer on the island also continues to increase [14]. A higher percentage of patients with HPS reported often or always engaging in each sun protection behavior when compared to the average Puerto Rican adult except for wearing a hat which was comparable [32, 33]. This is a potential area for improvement particularly since most skin cancers reported occurred in the head and neck.

Higher income was significantly associated with an increased frequency of sunscreen use and a third of patients reported not using sunscreen frequently because it was too expensive. This is consistent with previous studies which have found that those who are employed and with private insurance report more engagement in sun protection behaviors [32]. Sunscreen price as a barrier to use may be overcome by local and national recommendations for insurers to cover sunscreen for the underserved. A study by Queen and co-workers in the pediatric population showed that sunscreen coverage in the New York Medicaid population can be a cost-effective intervention to preventing skin cancer [34]. Similar findings have been reported in subtropical areas regarding sunscreen coverage by government plans [35, 36].

We also assessed access to dermatologic care. The majority had a provider they were able to visit regularly. Those without regular dermatologic care expressed the desire to obtain it demonstrating that expanding access is a worthwhile priority in this population. Only area of residence had a significant effect on access with those in urban areas being less likely to report difficulties seeing a provider. This could explain why time and transportation were among the most reported barriers. This is consistent with nationwide studies showing dermatologists are unevenly geographically distributed, with most in dermatologist-dense areas [37]. Potential solutions might include tele-dermatology which has been shown to increase access to dermatologic care particularly among Medicaid-insured and poor urban and rural populations [38–40]. Torres Olan and co-workers reported on multidisciplinary clinics for HPS patients on the island in the Southwest region [41]. The continuation and expansion of these clinics may also increase access.

Finally, we sought to examine the understanding of skin cancer. All patients were aware they are at an increased risk. This prompted the vast majority to frequently examine their skin which is encouraging given that Niu et al. found that in Puerto Rican adults skin cancer knowledge had the strongest association with sun protection behaviors [32].

This study has several strengths including an expansive questionnaire addressing several areas of dermatologic care, a telephone format which ensured patient understanding, a diagnosis of HPS based on genetic analysis, and the geographic diversity of participants. However, it is also subject to several limitations. First, the information is self-reported and although patients were asked to refer to diagnoses given by a physician there is a potential for wrongful or unclear reporting of diagnoses. Further studies should involve a clinical examination by a dermatologist. Finally, given the rarity of the disease, it is difficult to achieve a large sample size, however, a larger study would allow for more robust statistical analysis particularly for assessing differences between subtypes of HPS.

This study provides an important overview of the most common self-reported skin conditions among patients with HPS in P.R. Most notably, a high prevalence of cutaneous malignancies was reported. The study results stress the need for adequate dermatologic care and the development of interventions to promote sun protection behaviors and skin cancer prevention.

## Data Availability

All data generated or analyzed during this study are included in this article. Further enquiries can be directed to the corresponding author.
